# Biological control of an agricultural pest protects tropical forests

**DOI:** 10.1038/s42003-018-0257-6

**Published:** 2019-01-07

**Authors:** K. A. G. Wyckhuys, A. C. Hughes, C. Buamas, A. C. Johnson, L. Vasseur, L. Reymondin, J. -P. Deguine, D. Sheil

**Affiliations:** 1 Institute of Applied Ecology, Fujian Agriculture & Forestry University, Fuzhou, Fujian 350002, People’s Republic of China; 2 China Academy of Agricultural Sciences CAAS, Beijing 100193, People’s Republic of China; 3 University of Queensland, Brisbane 4072, QLD Australia; 4 Zhejiang University, Hangzhou, Zhejiang 310058 People’s Republic of China; 5 Xishuangbanna Tropical Botanical Gardens, China Academy of Sciences CAS, Xishuangbanna, Yunnan 666303 People’s Republic of China; 6 Department of Agriculture (DoA), Ministry of Agriculture and Cooperatives, Bangkok 10900, Thailand; 7 Charles Sturt University, Orange, NSW 2800 Australia; 8 Brock University, St. Catharines, ON L2S 3A1 Canada; 9 International Center for Tropical Agriculture CIAT, 100000 Hanoi, Vietnam; 10 CIRAD, UMR PVBMT, F-97410 St Pierre, La Réunion, France; 11 Norwegian University of Life Sciences, P.O. Box 5003, 1432 Ås, Norway

## Abstract

Though often perceived as an environmentally-risky practice, biological control of invasive species can restore crop yields, ease land pressure and thus contribute to forest conservation. Here, we show how biological control against the mealybug *Phenacoccus manihoti* (Hemiptera) slows deforestation across Southeast Asia. In Thailand, this newly-arrived mealybug caused an 18% decline in cassava yields over 2009–2010 and an escalation in prices of cassava products. This spurred an expansion of cassava cropping in neighboring countries from 713,000 ha in 2009 to > 1 million ha by 2011: satellite imagery reveals 388%, 330%, 185% and 608% increases in peak deforestation rates in Cambodia, Lao PDR, Myanmar and Vietnam focused in cassava crop expansion areas. Following release of the host-specific parasitoid *Anagyrus lopezi* (Hymenoptera) in 2010, mealybug outbreaks were reduced, cropping area contracted and deforestation slowed by 31–95% in individual countries. Hence, when judiciously implemented, insect biological control can deliver substantial environmental benefits.

## Abstract (web summary)

Kris Wyckhuys et al. show how introduction of the host-specific natural enemy *Anagyrus lopezi* reduced outbreaks of an invasive insect pest and helped slow deforestation by 31–95% across Southeast Asia. This study illustrates the environmental benefits of ecologically-based crop protection in agricultural settings.

## Introduction

The UN Sustainable Development Goals of 2030 Agenda aim to end malnutrition and poverty while preventing biodiversity loss^1^. These goals place competing demands on land that are not readily reconciled^2–4^. For example, agricultural expansion serves many fundamental needs but often results in the clearing of forests with negative consequences for biodiversity, freshwater, and atmospheric composition^5,6^. Given the need to reconcile such competing demands on land, we must identify and promote all appropriate options including those that are often disregarded, such as arthropod biological control. Invasive species, including many agricultural pests, constrain the production of food and other commodities^7^, and often impose additional costs such as the disruption of ecosystem services (e.g., nutrient cycling), damage to infrastructure or increased disease in humans^8^. Since the late 1800s, more than 200 invasive insect pests and over 50 weeds across the globe have been completely or partially suppressed through biological control, often with favorable benefit:cost ratios (ranging from 5:1 to > 1000:1)^9,10^. Modern biological control, centered on a careful selection and subsequent introduction of a specialized natural enemy (obtained from the pest species’ region of origin), can offer an effective solution for invasive species problems^11^. This approach is particularly useful in smallholder farming systems in the tropics, as biological control is self-propagating and requires little involvement from local stakeholders^12^. Nonetheless there are risks, as exemplified by few (poorly selected) control agents that have subsequently become major problems themselves, such as the cane toad *Buffo marinus* L. or the weevil *Rhinocyllus conicus* Frölich^13,14^. As a consequence, despite significant improvements in risk assessment and management over the past three decades, concerns often obscure the potential benefits and result in biological control being avoided when it could be valuable^13^. While the failures of the last century appear well known, recent success stories require wider recognition. Our goal here is to present one such story.

Cassava, *Manihot esculenta* Crantz (Euphorbiaceae), is a globally important source of starch, a food staple for vulnerable rural populations in several Asian countries, and a base for the production of food products, animal feed, ethanol, and household items^15^. In Southeast Asia, cassava is cultivated on nearly 4 million ha and extensively traded. It is grown in small-scale diversified systems by smallholders as well as in large plantations operated by agro-industry. In late 2008, the invasive mealybug, *Phenacoccus manihoti* Matile-Ferrero (Hemiptera: Pseudococcidae) was first detected in Thailand’s cassava crop. Upon its arrival in Asia, it spread to its ecological limits (though confined by cassava cropping area)^16^, leading to an average 4.1 ton ha^−1^ reduction in crop yield in Thailand (from 22.7 to 18.6 ton ha^−1^), a 27% drop in the nation’s aggregate cassava production and an ensuing 162% increase in starch price^15^. One response was the 2009 introduction of the host-specific parasitoid wasp *Anagyrus lopezi* De Santis (Hymenoptera: Encyrtidae; originally native to South America) from Benin (West Africa), where it had suppressed *P. manihoti* throughout Africa following its introduction in 1981^17^. These wasps were released across Thailand from mid-2010 onward and were subsequently introduced into Lao PDR, Cambodia, and Vietnam (2011–2013). They established successfully and suppressed mealybug populations across the region^18^. This restored yields by 5.3–10.0 tonnes ha^−1^ (as assessed through manipulative assays) and helped stabilize the trade in cassava root, starch and substitute commodities (i.e., maize, wheat, potato)^15^.

In this study, we characterize how the cassava mealybug invasion and ensuing biological control can be associated with agricultural expansion and forest loss in mainland Southeast Asia. These forests include the most species-rich and biologically valuable habitats in the region^19,20^. We first conduct surveys to quantify the extent of parasitoid-mediated *P. manihoti* population suppression (*section—pest and parasitoid survey*). Second, we examine regional patterns of cassava cultivation and inter-country trade from 2009 to 2013 (*section—country-specific cassava production and trade trends*). Third, we contrast forest loss and cassava expansion over the above period (*section—country-specific trends in forest loss vs. cassava area growth*). Our work illuminates how scientifically underpinned biological control not only restores crop yields, but can also avert biodiversity loss and contribute to tropical forest conservation at a macro-scale.

## Results

### Regional pest and parasitoid survey

**Fig. 1:**
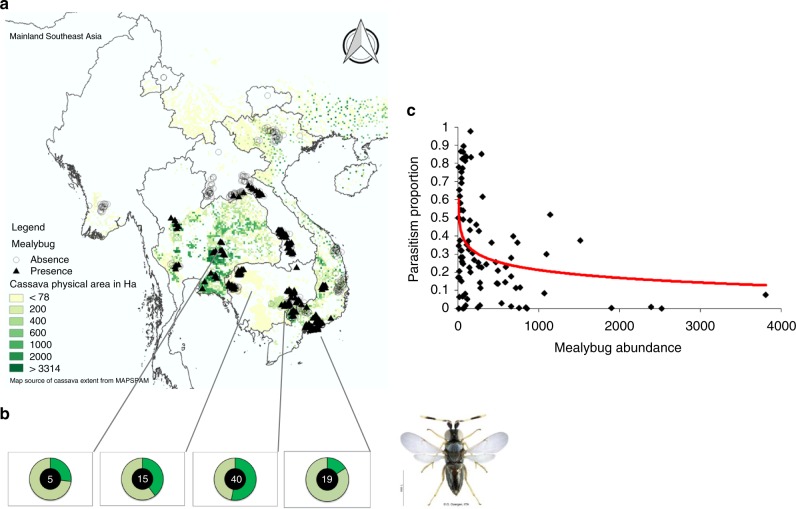
Map of Southeast Asia depicting *P. manihoti* geographical distribution, complemented with field-level *A. lopezi* parasitism and mealybug abundance records. In **a** green shading reflects the approx. 4 million ha of cassava cultivated regionally in 2005. **b** presents doughnut charts, indicative of the percent *A. lopezi* parasitism (as depicted by the dark green section over a light green background) at four selected sites. The number inside each doughnut reflects the number of fields sampled per locale. **c** presents the relationship between average *P. manihoti* abundance and *A. lopezi* parasitism level per field, for a total of 90 fields in which simultaneous recordings were done of mealybug infestation pressure and parasitism rate. Mealybug distribution maps were adapted from Wyckhuys et al.,^18^. Photograph *Anagyrus lopezi* (credit G. Goergen, IITA)

Our surveys, conducted across mainland Southeast Asia between 2014 and 2017 (i.e., 6–9 years and 5–8 years following the initial *P. manihoti* detection and *A. lopezi* introduction, respectively), showed that *P. manihoti* was present in 37.0% of the fields (*n* = 549) and comprised 20.8% abundance within a speciose mealybug complex^18^ (Fig. 1). Among sites, *P. manihoti* reached field-level incidence of 7.6 ± 15.9% (mean ± SD; i.e., proportion mealybug-affected tips) and abundance of 5.2 ± 19.8 individuals per infested tip. *Anagyrus lopezi* wasps were recorded in 96.9% of mealybug-affected fields (*n* = 97), at highly-variable parasitism rates. For example, in mid- to large-scale plantations parasitism rates ranged from 10.7 ± 10.6% (*n* = 20; Dong Nai, Vietnam) to 67.1 ± 20.8% (*n* = 22) in late dry season in Tay Ninh (Vietnam). In low-input, smallholder-managed systems (see methods), parasitism varied between 17.1 ± 14.8% (*n* = 18; Ba Ria Vung Tau – BRVT, Vietnam) to 46.7 ± 27.8% in central Cambodia (*n* = 10). Where *A. lopezi* was present, mealybug abundance was negatively associated with *A. lopezi* parasitism (ANOVA, *F*_1,84_ = 12.615, *p* = 0.001; Fig. 1^18^).

### Country-specific cassava production and trade

**Fig. 2:**
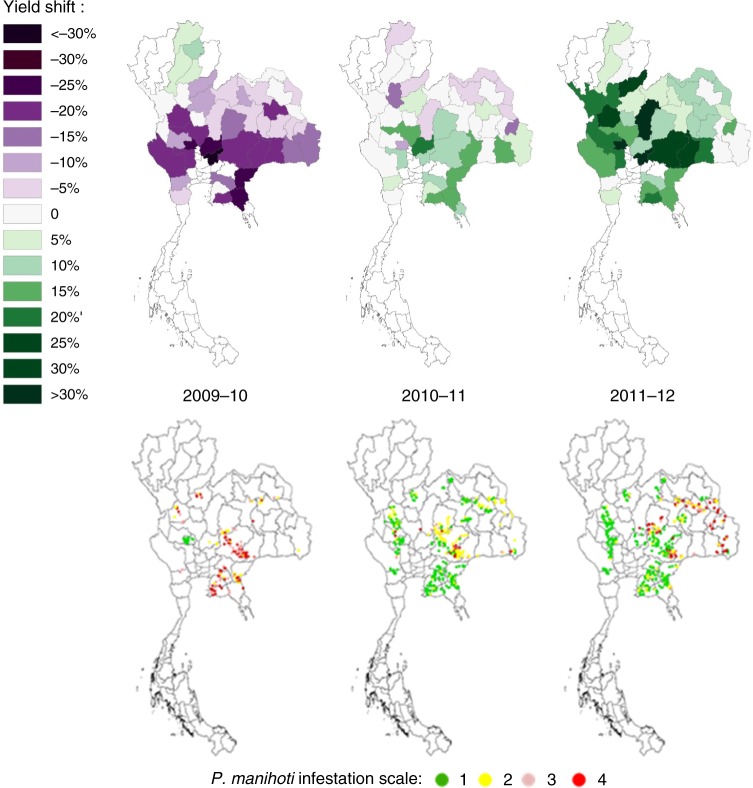
Yield recovery following biological control in Thailand’s cassava crop over 2009–12. Patterns are reflective of the country-wide cassava mealybug invasion (late 2008 onward) and ensuing biological control campaign. The upper panel reflects annual change in cassava crop yield (for a given year, in % as compared to the previous year) for a select set of provinces. In the lower panel, historical records of *P. manihoti* spatial distribution and field-level infestation pressure are shown over successive growing seasons (data facilitated through Thai Royal Government - Ministry of Agriculture & Cooperatives). The infestation scale ranks field-level *P. manihoti* attacks from low (1) to severe (4). Years indicated in the upper panel also apply to the lower panel. Maps in the upper panel were adapted from Wyckhuys et al.,^18^

In Thailand, cassava cropping area reached 1.3 million ha in 2009, and subsequently fell to 1.2 million (2010) and 1.1 million ha (2011). This followed the country-wide *P. manihoti* outbreak in 2009, and the ensuing yield losses and reduced cassava production. Time-lagged response is expected as cassava is a semi-perennial crop that is routinely harvested at 8–10 months of age, and planted at the onset of the rainy season^21^. Over the ensuing 2009–10 cropping season, province-level yields dropped by 12.6 ± 9.8% (area-weighted mean: −18.2%) and country-wide aggregate yields declined from 22.7 t ha^−1^ to 18.6 t ha^−1^ (Fig. 2). Regional production followed similar trends: total production across Vietnam, Myanmar, Lao PDR, and Cambodia dropped from 66.9 million tonnes in 2009 to 62.0 million tonnes in 2010 (Table 1). Yet, over 2009–2011, the volume of harvested cassava root in those countries increased substantially as cassava cropping area expanded (Supplementary Fig. 1 and 2).

**Table 1:** Inter-annual shifts in total cassava production (t), harvested crop area (ha), cassava root exports (t), and forest loss (ha) for four Southeast Asian countries, over a 2009–2013 time period

Country	Year	Total cassava production (%)	Harvested cassava area (%)	Cassava root exports (%)	Forest loss (%)
Lao PDR	2010				+ 80.4
	2011	+ 48.6	+ 56.1	+ 202.8	+ 90.0
	2012	+ 112.1	+ 120.5	+ 584.2	− 9.7
	2013	+ 150.8	+ 126.6	+ 942.7	− 11.5
Vietnam	2010				+ 207.0
	2011	+ 15.2	+ 12.1	+ 45.6	+ 90.5
	2012	+ 13.3	+ 10.8	+ 113.2	+ 24.6
	2013	+ 13.5	+ 9.3	+ 41.3	+ 35.0
Cambodia	2010				+ 168.9
	2011	+ 89.1	+ 82.7	+ 135.8	+ 138.5
	2012	+ 79.3	+ 66.6	+ 526.1	+ 94.2
	2013	+ 78.3	+ 61.1	+ 246.6	+ 241.8
Myanmar	2010				+ 104.8
	2011	+ 20.2	+ 25.7	−^a^	− 77.2
	2012	− 4.8	+ 0.3	–	− 41.2
	2013	− 13.7	− 3.1	–	− 25.0

For the first three parameters, percent annual change is calculated against a 2010 baseline, while yearly deforestation levels are compared with a 2009 baseline. Patterns are reflective of the 8–10 month time lag between cassava crop establishment and harvest. Forest loss records as extracted from the Terra-i platform

^a^No data available

From 2009 to 2012 regional trade in cassava-based commodities shifted, as Thailand’s import of cassava products (i.e., roots, chips, and pellets) increased by 153% and starch by 1575%, and Vietnam exported larger volumes of those products to China. In 2009, Thailand imported 1126 tonnes of cassava products from Lao PDR and 322,889 tonnes from Cambodia, and Vietnam’s exports equaled 2.09 million tonnes. By 2012, those country-level exports had risen up to 526–584% (Table 1). Over this period, there was a regional increase in cassava cropping area from 713,000 ha (2009) to > 1.02 million ha by 2011 (Supplementary Figs. 1 and 2). In all countries except Lao PDR, cropping area was largest in 2011 (Supplementary Fig. 3). By 2013, cassava area contracted and Thailand’s import trade of cassava products and starch dropped by 42.3–83.5% compared to 2012.

### Country-specific forest loss vs. cassava area

**Fig. 3:**
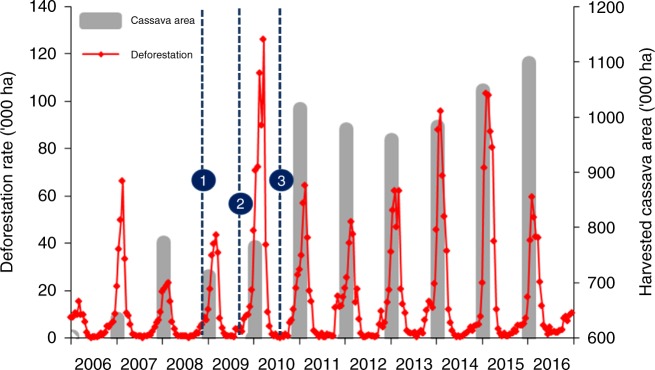
Near real-time deforestation patterns relate to the annual increase in (harvested) cassava area over a 2006–2016 time period. Satellite-derived biweekly deforestation rates are contrasted with the yearly, aggregate increase in harvested cassava area for Lao PDR, Vietnam, Cambodia, and Myanmar. Trends mirror the contribution of the semi-perennial cassava crop (harvested at 8–10 months of age) to deforestation at a regional scale. Patterns cover **a** The late 2008 invasion and subsequent regional spread of *P. manihoti* (event # 1), **b** The initial introduction of *A. lopezi* from Benin, West Africa (event #2), and **c** Nation-wide parasitoid release in cassava fields across Thailand (event # 3)

Regional deforestation surged in 2010 with an annual net loss of 653,500 ha as compared to 278,900 ha during the preceding year (Terra-i; Fig. 3). At both regional and country-specific level, this enhanced deforestation (concentrated during the November–March dry season) partially mirrored the increased volume of harvested cassava over 2011 (for an 8–10 month-long crop; see above) (Fig. 3 and Supplementary Fig. 3). In 2010, Terra-i estimated total forest loss up to 207% higher than in 2009 (Table 1), with deforestation peaking during early 2010 at 20,181 ha per week in Cambodia, 17,015 ha per week in Vietnam, and 51,284 ha per week in Myanmar (Supplementary Fig. 3). Peak deforestation rates during the 2010 dry season were a respective 388% (Cambodia), 608% (Vietnam), 185% (Myanmar) higher than those in 2009, and 2011 rates for Lao PDR represented a 330% increase. By 2011, peak deforestation rates in Cambodia, Vietnam, and Myanmar had declined by 31.8–94.9% compared to 2010, while those in Lao PDR lowered by 50.5% in 2012 (Supplementary Fig. 3).

**Fig. 4:**
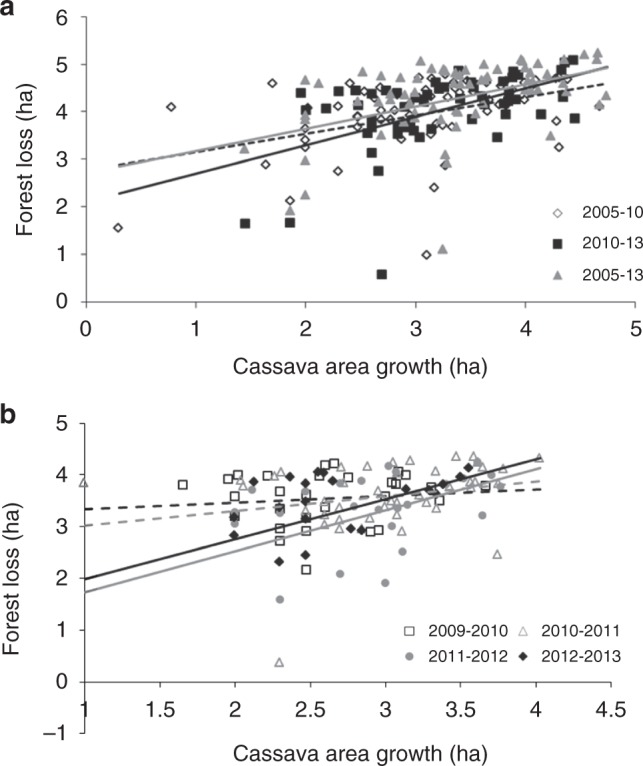
Regional and country-specific patterns in deforestation relate to growth of cassava cropping area over 2005–2013. **a** represents regional patterns, showing province-level cassava area increase (ha) in Vietnam, Cambodia, and Lao PDR as related to degree of forest loss (ha) over a 2005–10, 2010–2013, and entire 2005–2013 time frame. **b** contrasts annual forest loss against increase in (harvested) cassava area, for 40 different Vietnamese provinces. Both variables are log-transformed, and only certain regression lines in **b** reflect statistically significant patterns (ANOVA, *p* < 0.05; see text for further statistics). Data are exclusively shown for provinces and time-periods in which cassava area expansion was recorded. Dashed lines represent patterns for 2005–10 (**a**) and 2009–10, 2010–11 (**b**)

**Fig. 5:**
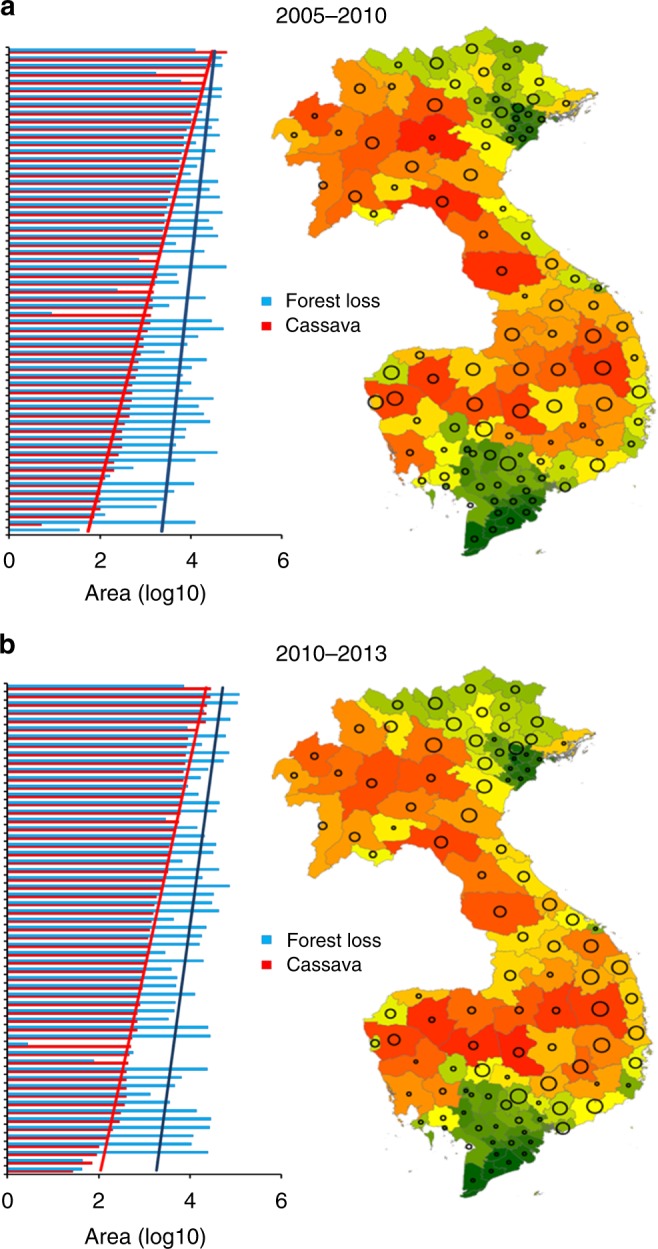
Forest loss relates to cassava area expansion across the Greater Mekong sub-region, over two distinct time-periods (i.e., 2005–2010, **a**; 2010–2013, **b**). Province-level deforestation and cassava area growth over particular time-periods are visualized for Lao PDR, Cambodia, and Vietnam, with bubble size depicting cassava area growth (ha) over that period and coloring reflecting level of forest loss (with increasing levels of forest loss indicated by colors ranging from green to red). Bar charts on the left contrast province-level (log-transformed) forest loss with cassava area increase at a regional level, exclusively for provinces in which cassava crop expansion was recorded over a particular time period

Examining patterns at a multi-country level, a significant association was recorded between (province-level, summed) deforestation and cassava area growth over 2005–2010 (ANOVA; *F*_1,61_ = 17.851, *p* < 0.001), over 2010–2013 (*F*_1,56_ = 20.547, *p* < 0.001), and over the entire 2005–2013 time period (*F*_1,65_ = 21.467, *p* < 0.001) (Figs. 4 and 5). For Vietnam specifically, province-level forest loss was positively related to the extent of (harvested) cassava area growth during 2011–2012 (*F*_1,24_ = 7.113, *p* = 0.013) and 2012–2013 (*F*_1,20_ = 4.603, *p* = 0.044), but not during 2009–2010 (*F*_1,27_ = 0.295, *p* = 0.591) or 2010–2011 (*F*_1,40_ = 2.863, *p* = 0.098). Similar patterns and associations were recorded for Cambodia for 2005–2010 and 2010–2012 (Supplementary Fig. 4). In cassava crop expansion areas, the extent of cassava area increase was thus directly associated with the degree of forest loss— revealing cassava to be an important, but not exclusive, driver of forest loss. Other drivers of importance might have been crops such as maize, rubber, or pulp/paper crop establishment. Since 2014, deforestation in Cambodia and Vietnam has continued (Supplementary Fig. 3), likely reflecting continuing growth of China’s demand for cassava products among others.

## Discussion

We have shown how the 2008 *Phenacoccus manihoti* invasion in Thailand contributed to a > 300,000 ha increase in cassava cropping area in Cambodia, Lao PDR, Vietnam, and Myanmar, to make up for a shortfall in supply and an 138–162% surge in cassava prices. More specifically, mealybug-induced yield shocks contributed to price surges in Thailand^15^ and coincided with 136–584% inter-annual increases in import flows of cassava products (Supplementary Fig. 2). Given the multiple determinants of commodity trade, we do recognize a difficulty to unambiguously identify drivers of the observed trade shifts and thus infer causality. Yet, in the years following the 2008 *P. manihoti* invasion, inter-country cassava trade significantly contributed to deforestation. The mealybug invasion equally prompted broad and recurrent use of systemic insecticides in Thailand (Supplementary Notes and Supplementary Fig. 5), with potential impacts on biodiversity, human health^22^ and ecosystem functioning^23,24^, including interference with *P. manihoti* biological control (Wyckhuys et al., unpublished). Given the importance of cassava for local smallholder families, the changes in crop productivity (e.g., total crop loss in 2009–2010 in parts of Thailand and western Cambodia) also had marked socio-economic impacts on rural livelihoods including declines in farmer income. The introduction of *A. lopezi* not only provided an environmentally-safe alternative for *P. manihoti* control but also allowed Thailand’s cassava production to recover, helped stabilize cassava trade, averted the need for insecticides in neighboring countries, and reduced cassava-driven forest loss in the region.

Demand for cassava is an important driver of land-use pressure and forest loss in the Greater Mekong sub-region, yet it is not the only one, and the *A. lopezi* introduction alone thus will not avert future deforestation. Other drivers of forest loss are the establishment of oil palm, pulp and paper plantations, rubber and food crops such as maize^25^; crops that are cultivated across tropical Asia through significant engagement from agro-enterprises^25,26^, with their actions regularly affected by ‘‘place-less’’ incentives (e.g., varietal improvements)^27,28^, (foreign-based) consumer demand and associated market forces^15^, or soil fertility loss, e.g., for cassava in upland settings^29^. Yet, during 2010–2012, our analyses revealed the marked role of cassava area growth in triggering deforestation at a multi-country level. To stabilize the forest margin, a multi-functional ‘‘landscape approach’’ and a systematic analysis of the multiple drivers of land-use change will thus be necessary^30^. Also, in order to gauge the inherent capacity of cropping systems to absorb (or recover from) disturbances such as the *P. manihoti* attack, indices can be adopted that reflect ‘‘ecosystem resilience.’’ Through use of those indices, agro-industry can simultaneously contribute to agricultural sustainability and biodiversity conservation^31,32^, while such ‘‘resilience’’ indices could be employed by different actors to further encourage good practice. By stabilizing cassava yields and alleviating pressure on land and dependence on synthetic insecticides, biological control supports agricultural intensification, safeguards farm profitability and spares land for conservation^4,33^. Nonetheless, while such land-sparing activities are valuable, these are insufficient to achieve conservation in the long-term without suitable policies, planning, governance arrangements, funding, and implementation^30,34^.

Several factors contributed to the success of the mealybug biological control program. These include early detection^35^; proper identification of the pest^36^; availability of and unrestricted access to an effective host-specific parasitoid^37^, and decisive action with private-sector involvement, including through the Thai Tapioca Development Institute (TTDI). These factors allowed an effective program to be swiftly planned, assessed, and implemented^38,39^, without the benefits of biological control being obscured by its risks. Although some cases have justifiably blemished the reputation of arthropod biological control, current practices, and safeguards minimize such risks^13,40^. Our study also helps put such risks into perspective, as the rapid *A. lopezi* introduction and field-level release proved essential to alleviate the disruptive impacts of *P. manihoti* attack^35,41^. The advantage of deploying an exotic, specialist parasitoid that had co-evolved with the target pest is further accentuated by Thailand’s initial effort to employ laboratory-reared native natural enemies—e.g., the generalist lacewings *Plesiochrysa ramburi* (Schneider) and *Mallada basalis* (Walker). Mass-releases of these mobile generalist predators in mealybug infestation hotspots not only proved rather ineffectual and uneconomic, but likely caused certain (transient) impacts on non-target species in nearby non-crop habitats. Hence, the risks of introducing a host-specific natural enemy (or ‘‘dietary specialist’’) such as *A. lopezi* were substantially lower than the risk of inaction.

The benefits gained through the *A. lopezi* release equally need to be viewed in light of the multi-faceted ecosystem impacts of invasive species^42,43^, and the environmentally-disruptive actions that are regularly taken for their mitigation^44,45^. Our study illustrates how an invasive pest can lead to substantial loss of forest^46^ and thereby accelerate species loss (including of endemic natural enemies) and extinctions^25,47^, and how scientifically guided biological control can offer an environmentally benign tool to resolve those impacts^11^. By concurrently highlighting the harmful and beneficial impacts of *P. manihoti* and *A. lopezi*, respectively, our work shows how biological control constitutes a practical ‘‘win-win’’ solution that tackles invasive species mitigation, biodiversity conservation and profitable farming. Collaboration between conservation biologists and crop protection scientists can thus be beneficial to balance farmer realities on the ground (e.g., pest control) with biodiversity conservation, while maximizing the contribution of off-farm habitat to field-level biological control^48^.

Biological control requires a reassessment by all those responsible for achieving a more sustainable world^2–4,49,50^. While invasive species undermine many of the UN Sustainable Development Goals^1,8,51^, the benefits of biological control are routinely disregarded^13,50^. Although an objective appraisal of risks remains essential, an equivalent recognition of the benefits is also warranted. When used with established safeguards^13^, biological control can resolve or reduce the problems caused by invasive species^11^ and helps ensure crop protection benefits not only farmers’ pocket^39^, but also the environment.

## Methods

### Pest and parasitoid survey

Insects were surveyed in 549 cassava fields in Myanmar, Thailand, Lao PDR, Cambodia, Vietnam, and southern China, from early 2014 until mid-2015, using standard protocols (see ref. ^18^). Briefly, 8–10-month-old fields in the main cassava-growing provinces of each country were selected with assistance from local plant health authorities, with sites located at least 1 km apart and within easy reach by vehicle. Surveys were conducted January–May 2014 (dry season), October–November 2014 (late rainy season) and January–March 2015 (dry season). Locations were recorded using a handheld GPS (Garmin Ltd, Olathe, KS). Five linear transects were established per field (or site), departing from positions along an X sampling pattern covering the entire cassava field. Ten consecutive plants were sampled along each transect, thus yielding a total of 50 plants per site. Each plant was assessed for the presence and abundance (i.e., number of individuals per infested tip) of *P. manihoti*. In-field identification of *P. manihoti* was based on morphological characters, and samples were equally transferred to the laboratory for further taxonomic confirmation. For each site, average *P. manihoti* abundance (per infested tip) and field-level incidence (i.e., proportion of *P. manihoti*-infested tips) was calculated.

To contrast local *P. manihoti* infestation pressure with *A. lopezi* parasitism rates, we sampled during 2014 and 2015 at a random sub-set of mealybug-invaded sites in different provinces in Thailand (*n* = 5), Cambodia (*n* = 10, 15 per province), and southern Vietnam (*n* = 18, 20, 22). In doing so, samples were obtained from both smallholder-managed, diversified systems (i.e., 1–2 ha in size) and from mid- to large-scale plantations (i.e., at least 5–10 ha in size). Sampling for *A. lopezi* parasitism consisted of collecting 20 mealybug-infested cassava plant tips at each site, which were transferred to a field laboratory for subsequent parasitoid emergence. Upon arrival in the laboratory, each cassava plant tip was examined, predators were removed and *P. manihoti* counted. Next, tips were placed singly into transparent polyvinyl chloride (PVC) containers, covered with fine cotton mesh. Over the course of 3 weeks, containers were inspected daily for emergence of *A. lopezi* parasitic wasps. Parasitism levels of *A. lopezi* (per tip and per site) were calculated. Next, for sites where *A. lopezi* was found, we analyzed field-level *P. manihoti* abundance with *A. lopezi* parasitism rate with linear regression (see also ref. ^18^). The latter analysis can reflect the degree of parasitoid control over its host, and give an initial appreciation of the extent of *A. lopezi*-mediated mealybug population suppression. Variables were log-transformed to meet assumptions of normality and homoscedasticity, and all statistical analyses were conducted using SPSS (PASW Statistics 18).

### Country-specific cassava production and trade trends

To assess how mealybug invasion and ensuing parasitoid-mediated cassava yield recovery affected cassava production and trade, we examined country-level production and inter-country trade for cassava-derived commodities. More specifically, we contrasted cassava yield and production trends with inter-country trade flows over periods spanning the 2008 *P. manihoti* invasion, the 2009 *A. lopezi* introduction into Thailand and the subsequent (natural, and human-aided) region-wide distribution of *A. lopezi* (mid-2010 onward). Our assessments detailed shifts in cassava production (harvested area, ha) and yearly trade flows (quantity) of cassava-derived commodities into Thailand from neighboring countries within the *P. manihoti* invaded range.

Crop production statistics for Thailand were obtained through the Office of Agricultural Economics (OAE), Ministry of Agriculture & Cooperatives (Bangkok, Thailand). Furthermore, country-specific patterns of cassava production (harvested area, ha) and yield (t ha^−1^) were obtained for Vietnam, Myanmar, Lao PDR, and Cambodia via the FAO STAT database (http://www.fao.org/faostat/). To assess structural changes in the inter-country trade of cassava-derived commodities, we extracted data from the United Nations Comtrade database (https://comtrade.un.org/). Over a 2006–2016 time period, we recorded the following evolutions in terms of quantity (tonnes): global annual imports of cassava-derived commodities to Thailand (reporting) and China, from ‘‘All’’ trade partner countries. More specifically, we queried the database for bilateral trade records of three cassava-derived commodities and associated Harmonized System (HS) codes: “Cassava whether or not sliced—as pellets, fresh or dried” (71410), “Tapioca & substitutes prepared from starch” (1903), and “Cassava starch” (110814). Given the occasional inconsistencies in country-reported trade volumes or values in either FAO STAT or Comtrade databases, cross-checks were made with databases from the Thai Tapioca Starch Association (TTSA) and corrections were made accordingly.

### Country-specific trends in forest loss vs. cassava area growth

To infer the likely impact of cassava area growth on forest loss in different Southeast Asian countries, we obtained data from both a near-real-time vegetation monitoring system, Terra-i (https://www.terra-i.org) and deforestation data from Global Forest Watch^52^ (https://www.globalforestwatch.org/). Terra-i relies upon satellite-derived rainfall and vegetation data obtained through TRMM sensor data (Tropical Rainfall Monitoring Mission) and MODIS MOD13Q1, respectively, to detect deviations from natural vegetation phenology patterns that cannot be explained by climatic events. More specifically, Terra-i adopts computational neural networks to detect how vegetation vigor behaves at a given site over a period of time in relation to observed rainfall, and thus identifies certain anomalies while accounting for the effects of drought, flooding and cloud cover or other image ‘‘noise.’’ Changes in vegetation greenness at the landscape level are recorded on the Terra-i platform on a biweekly basis. Terra-i outputs have been validated through comparison with the Global Forest Change data and the PRODES system in Brazil. These datasets showed similar values as the average KAPPA coefficient at 0.96 ± 0.004. Furthermore, the average recall value for detection of events with an area of 90% to 100% of a MODIS pixel is of 0.9 ± 0.05, which shows that Terra-i detects the large-size events. However, an average recall of 0.28 ± 0.13 has been observed when the event size is about 10% of a MODIS pixel, showing a limitation of Terra-I to detect smaller size tree cover clearance. Country-level deforestation statistics over a 10-year time period were extracted from this platform for Lao PDR, Myanmar, Vietnam, and Cambodia, and data were compiled on a province-level for each year from 2005 to 2013.

Next, yearly province-level records of cassava (harvested) area were compiled for each of the different countries by accessing FAO STAT, the Cambodia 2013 agriculture census and primary datasets as facilitated through national authorities and the International Food Policy Research Institute (IFPRI), Washington DC, USA. For Lao PDR, province-level records were only available on cultivated area of all root crops combined. Here, we assumed that major inter-annual changes in harvested area of root crops in Lao PDR can be ascribed to cassava as other locally important root crops, such as yam and sweetpotato are mostly grown for subsistence purposes and are less subject to major inter-annual area shifts. No continuous yearly datasets on local cassava cultivation area were available for Cambodia, and no province-level cassava cultivation records could be accessed for Myanmar. Because of these variations in data availability, some analyses were carried out over different periods (see below).

To quantify the extent to which forest loss was related to cassava area expansion, two types of analyses were conducted. First, we used linear regression to relate province-level increases in harvested cassava cropping area with forest loss during that same period for all countries (i.e., Cambodia, Lao PDR, Vietnam), over three different time frames: 2005–2010, 2010–2013, and 2005–2013. Second, as complete annual records on (province-level) cassava cultivation were available for Vietnam, linear regression analysis allowed annual province-level trends in forest loss to be related to cassava expansion for individual years (2009–2013). To meet assumptions of normality and heteroscedasticity, data were subject to log-normal (for cassava area records) or rank-based inversed normal transformation (for deforestation rates and records). All statistical analyses were conducted using SPSS (PASW Statistics 18).

## Supplementary information

Supplementary Information

## Data Availability

All data are made available in Dryad Digital Repository: 10.5061/dryad.1048v5p. Correspondence and requests for materials should be addressed to Kris A.G. Wyckhuys.
