# Supplementary material for: Biological control of an agricultural pest protects tropical forests
Source: Commun Biol. 2019 Jan 7;2:10. doi: 10.1038/s42003-018-0257-6 (PMC6323051; doi:10.1038/s42003-018-0257-6)
Supplement: Supplementary file 1 — Supplementary Information [file 42003_2018_257_MOESM1_ESM.pdf]

## Supplementary Information

**A**

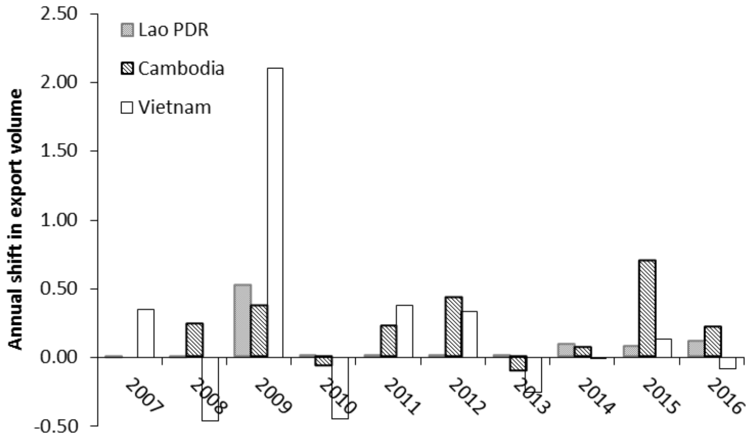


**B**

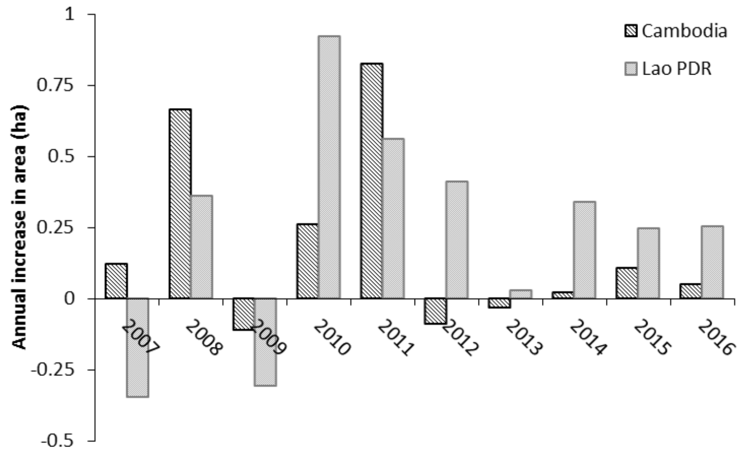


-

Supplementary Figure 1. Country-specific patterns in the annual proportional shift in (weighted) average export volume of cassava chips and pellets from Cambodia and Lao PDR (to Thailand) and Vietnam (to China) (*panel A*). *Panel B* shows annual proportional increase in harvested cassava area (ha) for Cambodia and Lao PDR.

**A** **B**

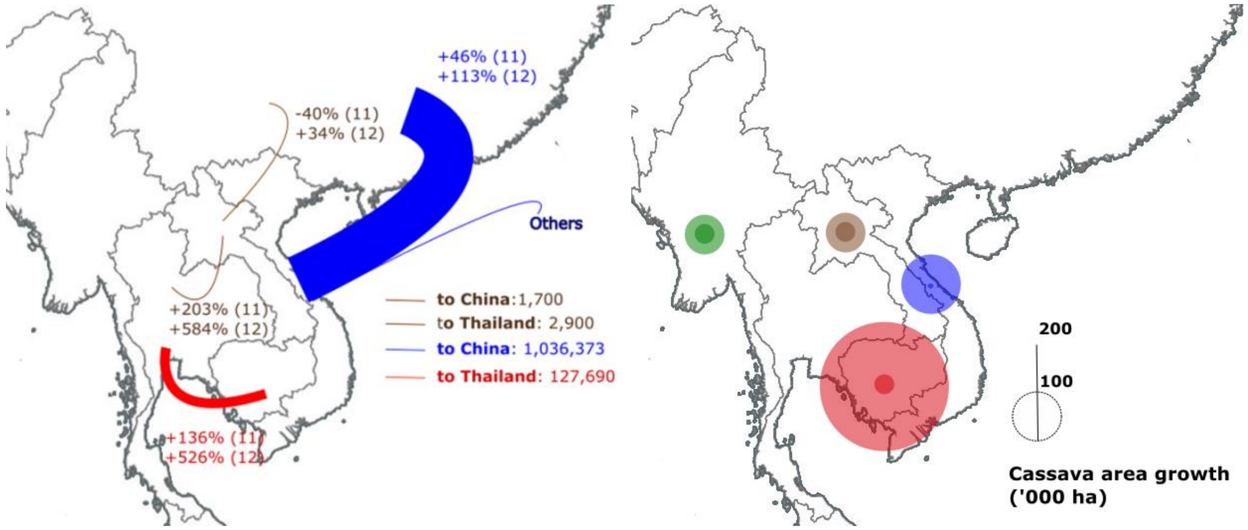


Supplementary Figure 2. Annual shifts in inter-country cassava trade mirror country-level expansion of cassava cropping area. In *panel A*, export volume is depicted of cassava roots, chips and pellets from Cambodia and Lao PDR (to Thailand and China) and Vietnam (to China). Thickness of the arrow reflects relative volume of traded cassava, and yearly increases in export volume are specified for 2010-2011, and 2011-2012 (A). *Panel B* depicts the annual rate of increase in harvested cassava area (ha) for individual Southeast Asian countries (except Thailand), from 2009 until 2011.

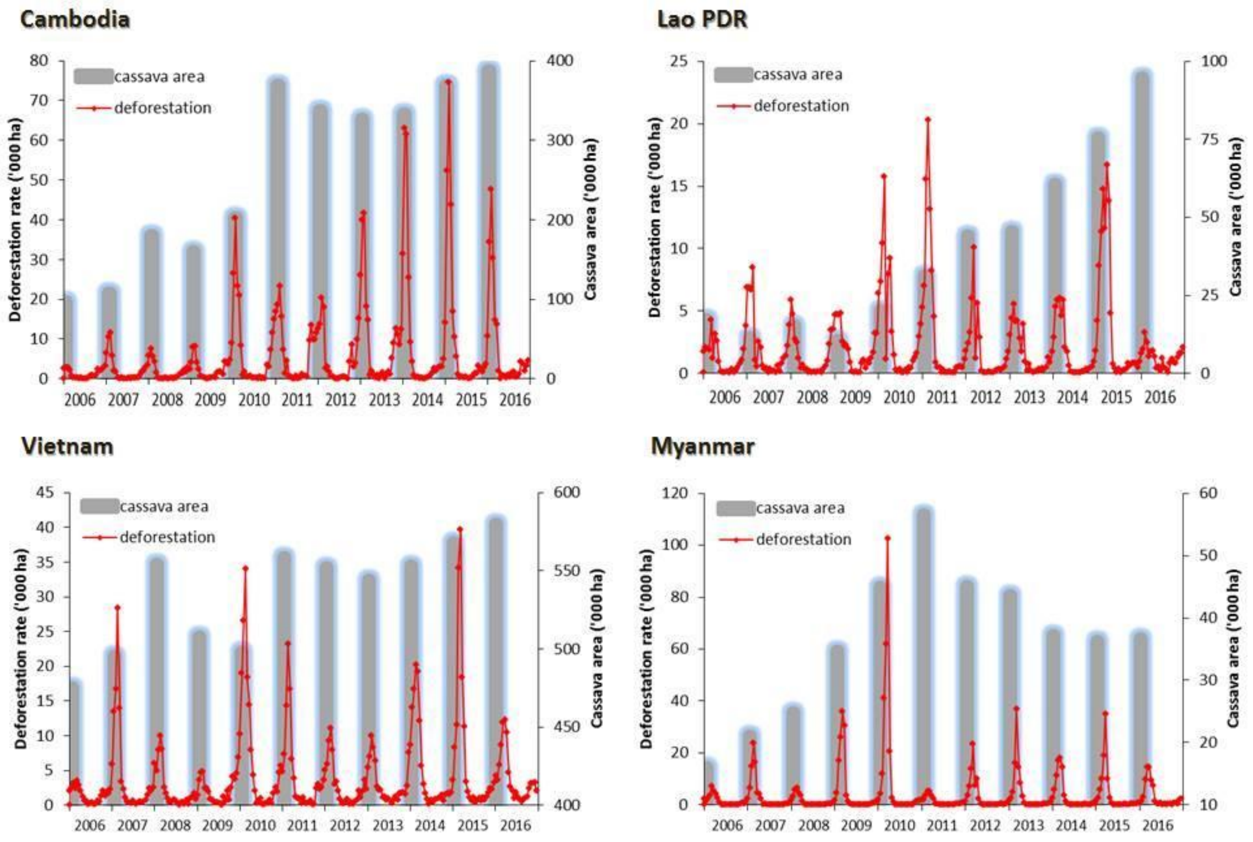


<u>Supplementary Figure 3</u>. Country-specific deforestation patterns as related to the annual increase in (harvested) cassava area over a 2006-2016 time period, covering the late 2008 invasion and subsequent continent-wide spread of *P. manihoti*, and the release of *A. lopezi* across Thailand in mid-2010.

A

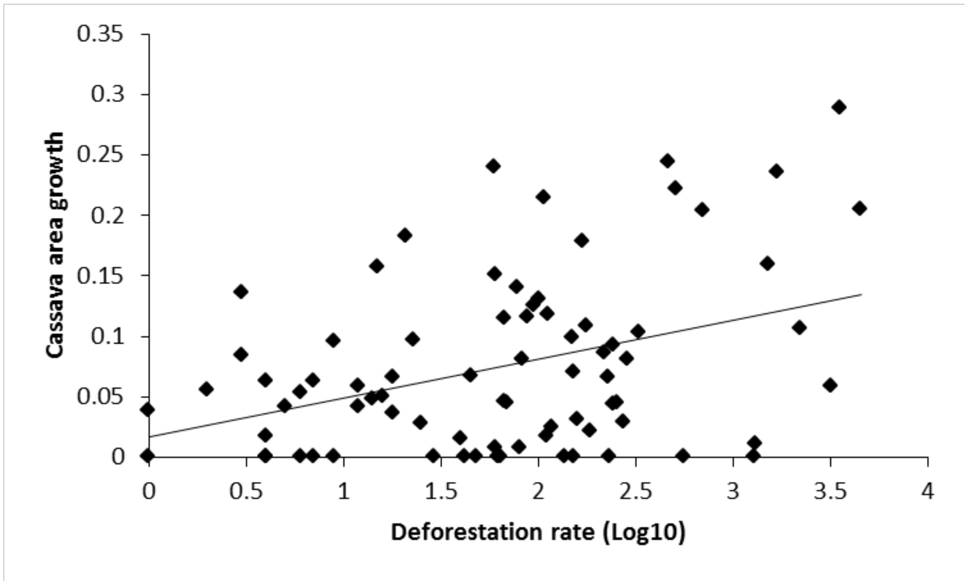


B

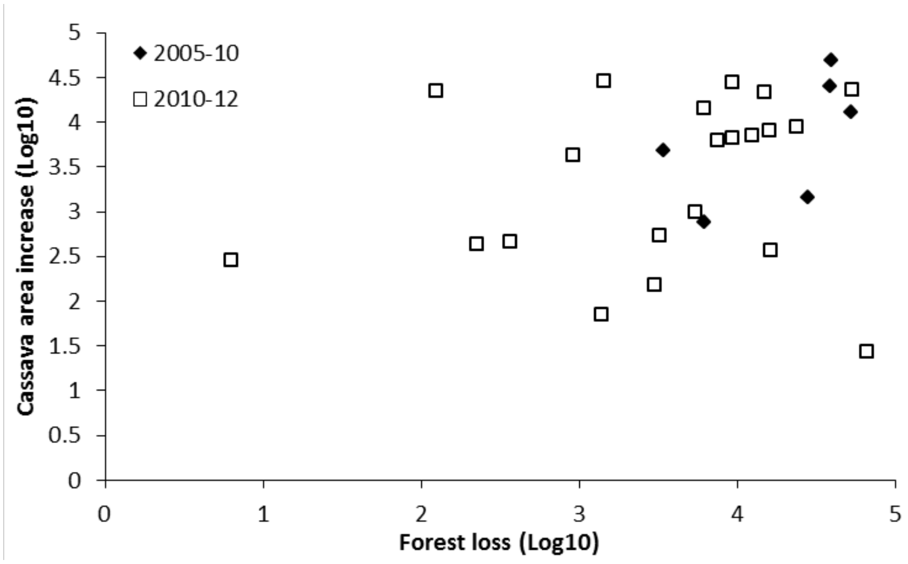


Supplementary Figure 4. Country-level cassava area-growth patterns, as related to local forest loss. Panel A depicts the proportional annual increase in (planted) cassava area as related to local degree of forest loss during the preceding year, for 40 different Vietnamese provinces over a 2008-2012 time period. *Panel B* represents province-level cassava area increase (ha) as related to degree of forest loss (ha), for 24 Cambodian provinces over a 2005-10 and 2010-12 time frame. Data are exclusively shown for provinces and time-periods in which cassava area expanded. Annual deforestation rates are log-transformed, and the regression line in *panel A* reflects a statistically significant pattern (ANOVA, $p < 0.05$).

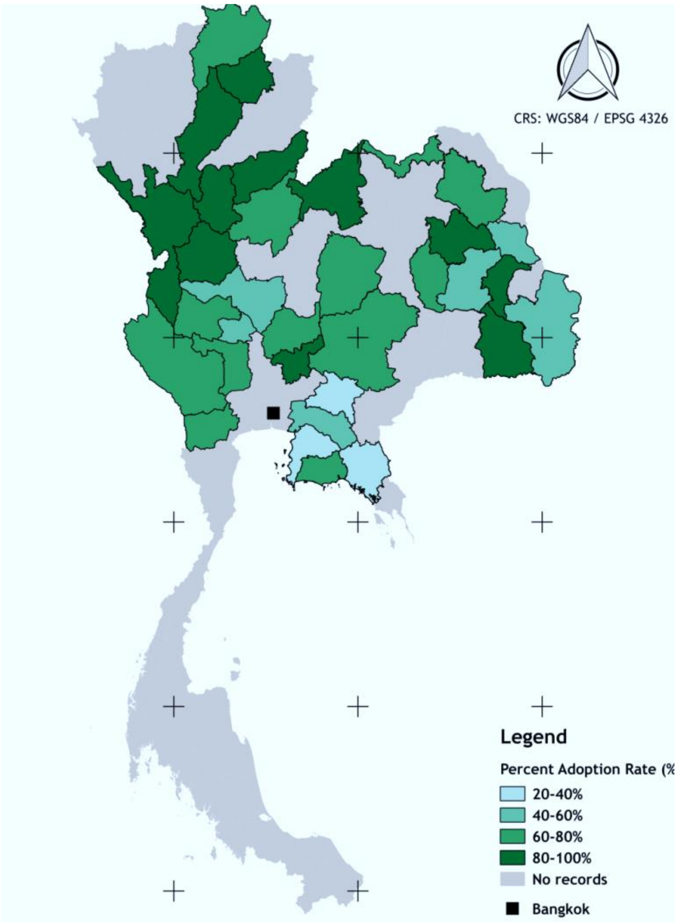


Supplementary Figure 5. Level of adoption of prophylactic insecticide dips amongst cassava farmers in 33 different provinces across Thailand, as recorded during mid-2014. Adoption levels are expressed as % of surveyed farmers in each province, within a nationwide survey of 2,500 growers (*n*= 20-348 per province). For provinces in grey, no data were obtained.

## Supplementary Methods

### Farmer adoption of insecticide use

From 2014 to 2016, extensive farmer surveys were conducted in Thailand. More specifically, household-level surveys were carried out using semi-structured questionnaires with open-ended questions, to optimally gauge farmer's knowledge and pest management behavior. One interview was done per household, following a person-to-person interview format. The questionnaire was pre-tested and revised prior to use at the national level. Surveys were entirely carried out by local officers from the Thai Department of Agricultural Extension (DoAE). At all sites, surveys were conducted by interviewers that were fluent in the local languages. Though survey instruments were designed for multiple purposes (e.g., Delaquis et al., unpublished), we only cover pest management activities in this study. For assessment of local pest management behavior, farmers were asked to freely enumerate knowledge and adoption of management practices for control of *P. manihoti*. Particular attention was paid to farmers' reported usage of (preventative) dips with neonicotinoid insecticides.

Farmer surveys were conducted in a total of 33 cassava-growing provinces over the course of 2014 (i.e., 6 years after the initial detection of *P. manihoti*). In each province, a variable number of farmers was interviewed by DoAE personnel, ranging from n= 20 (Roy-et, Payao) to n= 348 (Karnchanaburi), attaining a grand total of 2,505 cassava farmers in the national territory. Sample size was determined by local authorities, and is only partially reflective of the number of cassava growers in a given province. District-level adoption rates were pooled per province, and mapped at a national scale.

## Supplementary Notes

### Farmer adoption of insecticide use

In Thailand, 71.3% farmers (n= 2,505) used prophylactic dips with systemic insecticides for *P. manihoti* management (Supplementary Fig. 3). Regional adoption rates of insecticide dips ranged from 45.8% in eastern parts to 90.3% in northern areas of the country. Province-level rates of insecticide use were highest in Payao and Tak (100%; n= 20, 38 respectively), Lampang (98.0%; n= 49), Utaradit (96.1%; n= 26), Yasothorn (92.9%; n= 84) and Loei (91.9%; n= 123).
